# Impairment of the Zn/Cd detoxification systems affects the ability of *Salmonella* to colonize *Arabidopsis thaliana*

**DOI:** 10.3389/fmicb.2022.975725

**Published:** 2022-08-22

**Authors:** Sabina Visconti, Maria Luisa Astolfi, Andrea Battistoni, Serena Ammendola

**Affiliations:** ^1^Department of Biology, University of Rome Tor Vergata, Rome, Italy; ^2^Department of Chemistry, Sapienza University of Rome, Rome, Italy

**Keywords:** *Salmonella enterica*, transition metals, *Arabidopsis thaliana*, nutritional immunity, biofortification, *Salmonella*-host interaction, Zn transporters, Zn/Cd detoxification

## Abstract

*Salmonella* capacity to colonize different environments depends on its ability to respond efficiently to fluctuations in micronutrient availability. Among micronutrients, Zn, besides playing an essential role in bacterial physiology, is a key element whose concentration can influence bacterial survival in a particular niche. Plant colonization by *Salmonella enterica* was described for several years, and some molecular determinants involved in this host-pathogen interaction have started to be characterized. However, it is still unclear if Zn plays a role in the outcome of this interaction, as well established for animal hosts that employ nutritional immunity strategies to counteract pathogens infections. In this study, we have investigated the involvement of *Salmonella* Typhimurium main effectors of zinc homeostasis in plant colonization, using *Arabidopsis thaliana* as a model host. The results show that to colonize plant tissues, *Salmonella* takes advantage of its ability to export excess metal through the efflux pumps ZntA and ZitB. In fact, the deletion of these Zn/Cd detoxification systems can affect bacterial persistence in the shoots, depending on metal availability in the plant tissues. The importance of *Salmonella* ability to export excess metal was enhanced in the colonization of plants grown in high Zn conditions. On the contrary, the bacterial disadvantage related to Zn detoxification impairment can be abrogated if the plant cannot efficiently translocate Zn to the shoots. Overall, our work highlights the role of Zn in *Salmonella*-plant interaction and suggests that modulation of plant metal content through biofortification may be an efficient strategy to control pathogen colonization.

## Introduction

Over the last decades, it has emerged that some *Salmonella enterica* serovars, which are typically considered animal enteric pathogens, can also colonize plants (Schikora et al., 2008; Zheng et al., 2013; Zarkani and Schikora, 2021). *Salmonella* enters the plant primarily through stomata, by a process that requires both flagella and chemotaxis, and replicates inside plant tissues persisting in the apoplast (Kroupitski et al., 2009). Colonization of plant tissues enables *Salmonella* to evade the hostile environment of the leaf surface and reside in a protected and nutrient-rich niche. However, the internalization and persistence of such pathogens could be affected by plant growth conditions and by the availability of specific nutrients.

Transition metals are micronutrients that can drive the interaction between hosts and pathogens, being the key factors of the nutritional immunity strategies in mammals that modulate their availability for the invading microorganisms as a defense mechanism (Murdoch and Skaar, 2022). Metals, such as Zn, Fe, Cu, and Mn are essential nutrients for the organisms of every kingdom of life, playing roles in cellular metabolism, as cofactors of numerous enzymes, in the structural stability of macromolecules, and innate immunity mechanisms. For these reasons, adequate uptake of these essential elements must be ensured, but, at the same time, unnecessary intracellular metal accumulation must be avoided (Chandrangsu et al., 2017). They can be toxic over a certain threshold concentration, primarily due to mismetallation of proteins, where the native metal is replaced by the excess metal and modifies the structure or activity of the protein itself (Barwinska-Sendra and Waldron, 2017). Moreover, some metal ions can trigger harmful reactions, such as excess iron that can generate toxic radicals *via* the Fenton reaction. Thus, each organism must control the abundance of such elements through strictly regulated homeostatic mechanisms.

The infected host exploits both the pathogen’s need for an adequate transition metal recruitment by lowering their availability in the infected tissues and the toxicity of these elements over a certain concentration (Cerasi et al., 2013; Núñez et al., 2018). The latter is the case, for example, of macrophages, which employ Cu and Zn to kill bacteria by actively pumping these metals into the phagosomes (Sheldon and Skaar, 2019). Pathogens can also be targeted by metal accumulation on mucosal surfaces, where Zn can be mobilized by the host and interfere with the uptake of other essential metals (McDevitt et al., 2011; Djoko et al., 2015).

The ability of *Salmonella enterica* to colonize different niches also relies on strategies that allow facing metal availability fluctuations, from limiting to excess conditions. In adapting to Zn shortage, low and high-affinity Zn importers ensure an adequate supply for *Salmonella* coping with Zn restricted environments. The ZnuABC high-affinity Zn uptake system, whose expression is regulated by the Zn-sensing transcription factor Zur, is critical for full *Salmonella* virulence in different animal models. It has been shown that an impaired ability to acquire Zn can affect the intestinal and systemic colonization of the host, the ability to outcompete the resident microbiota in the inflamed gut, and the response to nitrosative stress in phagosome engulfed bacteria (Ammendola et al., 2007; Pasquali et al., 2008; Liu et al., 2012; Cerasi et al., 2013; Fitzsimmons et al., 2018). Evidence on the role of Zn detoxification systems in *Salmonella* interaction with the host is still limited. *Salmonella* responds to Zn excess mainly by ZntA, a P_1B_-type ATPase induced by ZntR, when intracellular free Zn exceeds the nanomolar threshold (Wang et al., 2012). It has been suggested that ZntA could be involved in *Salmonella* resistance to macrophage killing, together with ZitB, a cation exporter previously described in managing the physiological efflux of Zn during bacterial metabolism (Frawley et al., 2018; Huang et al., 2018).

In plants, mechanisms of nutritional immunity have been poorly explored. It is known that, in the interaction with phytopathogens, the plant responds by activating defense mechanisms relying on complex networks of signaling pathways that trigger transcriptional reprogramming, oxidative burst, and hormonal changes to counteract the pathogen attack (Berens et al., 2017; Birkenbihl et al., 2017). However, it has been proposed that the high concentration of Zn in the tissue of metal accumulating plants, e.g., *A. halleri*, could be a defense mechanism against the pathogens (Cabot et al., 2019).

In this work, we have investigated the role of ZntA and ZitB in the ability of *Salmonella enterica* serovar Typhimurium to colonize *Arabidopsis thaliana* seedlings. We have demonstrated that *Salmonella* faces an environment that induces the expression of the Zn detoxification systems and that the functionality of both Zn exporters is required for bacteria survival in plant tissue under Zn supplementation.

## Materials and methods

### Reagents

All chemicals used for solutions were purchased as ultrapure reagents from Sigma-Aldrich (Sigma-Aldrich Corporation, St. Louis, MO, United States). The antibiotics were dissolved as concentrated stock solutions, sterilized through filtration, stored at −20°C, and used at the following concentrations: kanamycin 50 mg L^−1^, chloramphenicol 30 mg L^−1^. Zinc Sulfate (ZnSO_4_), and Cadmium Acetate [Cd(CH_3_CO_2_)_2_] were prepared fresh before use by dissolving the powders in ddH_2_O as 0.5 M stock solutions.

### Bacterial strains and growth conditions

All *Salmonella* strains are derivatives of *Salmonella* Typhimurium ATCC14028s (STM). Strain SA182 (*znuABC::kan*) and strain SA395 (*zntA::kan*) carry a kanamycin cassette insertion in the *znuABC* and *zntA* coding sequence, respectively (Pasquali et al., 2008; Ammendola et al., 2014). Strain SA468 (*zitB::cam*) was obtained by one-step recombination protocol (Datsenko and Wanner, 2000) using a PCR fragment amplified on plasmid pKD3 with oligonucleotides oli-290 / oli-291 (Supplementary Table S1). Recombinants were checked by single-colony PCR with oligonucleotides K3 /oli-292 (Supplementary Table S1). The *zitB::cam* allele was transduced in a clean background by phage P22 HT 105/1 *int-201* generalized transduction, obtaining strain SA468. Strain SA469 (*zntA::kan zitB::cam*) was obtained by P22 transduction of the *zitB::cam* allele into strain SA395 as already described (Ammendola et al., 2016). Bacteria were routinely cultured in Luria Bertani broth (LB: Peptone 10 g L^−1^, Yeast extract 5 g L^−1^, NaCl 10 g L^−1^, and pH 7.5) at 37°C with aeration. For metal sensitivity assays, the chemical defined Vogel Bonner Minimal Medium (VBMM: anhydrous MgSO_4_ 0.04 g L^−1^, citric acid 2 g L^−1^, anhydrous K_2_HPO_4_ 10 g L^−1^, NaH_4_PO_4_ 3.5 g L^−1^, and glucose 2 g L^−1^) was employed. Growth curves were performed from overnight cultures grown in LB, diluted 1:500 in VBMM supplemented or not with metals in a 96-microwell plate (Greiner BioOne, Kremsmünster, Austria), incubated at 37°C for at least 15 h in a microplate reader (Sunrise TM, Tecan, Männedorf, Switzerland). Each condition was tested in triplicate and optical densities at 595 nm were recorded every hour.

### Plant lines and growth conditions

*Arabidopsis thaliana* Columbia-0 (Col-0) comes from a laboratory collection. The *hma2-4 hma4-2* line (hereafter referred to as *hma2/hma4*) was obtained from the Nottingham *Arabidopsis* Stock Centre and is a double mutant generated by crossing T-DNA insertional mutants SALK_034393 and SALK_050924. Plants were grown in half MS-agar medium (1/2 MS), containing MS 2.2 g L^−1^ (Duchefa Biochemie, Haarlem, Netherlands), 0.05% MES (Sigma-Aldrich Corporation, St. Louis, MO), 1.2% agar (BD, New Jersey, United States), and pH 5.7. Seeds were sterilized as previously described (Fiorillo et al., 2020) with 70% ethanol followed by 3% sodium hypochlorite in a 0.05% Tween-20 solution and extensively rinsed with sterile ddH_2_O. Seeds were then sown on 1/2 MS Petri dishes, followed by 48 h stratification treatment at 4°C to uniform germination. As the Zn concentration in 1/2 MS was 0.015 mmol L^−1^ Zn, in the case of Zn supplementation 0.135 mmol L^−1^ ZnSO_4_ was added to the medium, to reach a 0.15 mmol L^−1^ Zn concentration (1/2 MS + Zn). The plates were incubated vertically in the climate-controlled growth chamber at 22°C under a 16/8 light/dark cycle.

### Inoculation of *Arabidopsis thaliana* with *Salmonella* Typhimurium

*Salmonella* Typhimurium strains were inoculated overnight in LB, diluted 1:100 in fresh LB, and grown for 3 h at 37°C with aeration. At an OD_600_ of approximately 0.6, bacteria were diluted to 10^6^ CFU ml^−1^ in Potassium Phosphate buffer 0.5 mM, pH 7.4, and used for the inoculation of 10-day old *A. thaliana* seedlings. For this purpose, each seedling was wet with 0.05 ml of the bacterial suspension, withdrawing the excess liquid from the plate short after. A suitable dilution of the bacterial suspension was plated on LB-agar to count viable cells. The inoculated plants were incubated in the growth chamber at 22°C under a 16/8 h light/dark cycle. At different days post-inoculation (dpi), the shoots were cut and weighted using an analytical balance (sensitivity, 0.1 mg; mod BP1216; Sartorius, Göttingen, Germany). Each shoot was placed in 1.5 ml microcentrifuge tubes, surface-sterilized for 2 min in Phosphate Buffered Saline containing 0.1% SDS, 0.2% Tween 20, and 1% NaClO and extensively rinsed thoroughly in sterile ddH_2_O (Schikora et al., 2008). After adding 0.1 ml of 0.05 M MgCl_2_ and 20% glycerol, the shoots were mechanically homogenized using a sterile micropestel. The homogenates were opportunely diluted in phosphate-buffered saline (PBS) and plated on LB-agar to enumerate viable bacteria. For each sample, the number of colonies was related to the weight of the shoot and reported as CFU/mg of fresh weight (FW).

### Competition assays in *Arabidopsis thaliana*

The competition assays were performed following the same protocol used for single-strain inoculation experiments, except that the bacterial suspension used for plant challenge (input) was a 1:1 mixture of two different strains. An aliquot of the input mixture was plated on LB-agar, and 200 colonies were checked for antibiotic resistance to calculate the input ratio (strain A/strain B). At 3 dpi the shoots were removed, surface sterilized, and homogenized in 0.1 ml of 0.05 M MgCl_2_, 20% glycerol. The homogenates (outputs) were plated after suitable dilutions on LB-agar plates, and at least 200 colonies from each sample were checked for antibiotic resistance. The Competition Index (CI) for each sample was calculated as already described (Ammendola et al., 2007), by the formula [CI = input (strain A/strain B)/output (strain A/strain B)]. For each competition assay, the statistical significance was calculated by the Student *t*-test.

### RNA extraction and RT-qPCR

*Salmonella* Typhimurium ATCC14028 was inoculated in 2 ml LB supplemented or not with 1 mM Zn and grown until the mid-log phase (about OD_600_ = 0.5). According to the manufacturer’s instructions, an aliquot corresponding to approximately 10^7^ cells was employed for RNA extraction using the RNeasy mini kit (Qiagen GmbH, Hilden, Germany). The concentration and purity of the RNA were determined with a NanoDrop™ Lite Spectrophotometer (Thermo Fisher Scientific, Massachusetts, United States). For RT-PCR, three independent replicates for each experimental condition were prepared. RNA samples from plant-colonizing bacteria were prepared by total RNA extraction from STM inoculated *A. thaliana* seedlings. For this purpose, shoots were surface-sterilized and mechanically ground to a fine powder in liquid N_2_. RNA was extracted from 100 mg of powdered plant tissue using the RNeasy Plant Mini Kit RNA (Qiagen, Hilden, Germany) and stored at −80°C. The concentration and purity of the RNA were determined with a NanoDrop™ Lite Spectrophotometer (Thermo Fisher Scientific, Massachusetts, United States). RNA purified from *in vitro* grown bacteria and from plant-colonizing bacteria were used to prepare the cDNA, by retrotranscribing 1 μg of RNA for each sample using the PrimeScriptTM RT Reagent Kit (Takara Bio Inc., Shiga, Japan), according to the manufacturer’s instructions. The cDNAs obtained were stored at −80°C. For the RT-qPCR analysis SYBR GREEN dye (PCR Biosystems, London, United Kingdom) and the QuantStudio3 apparatus (Applied Biosystems, Waltham, Massachusetts, United States) were used. Bacterial gene expression levels from plant homogenates were compared to those from LB growth and calculated using the formula 2^-ΔΔCt^, where ΔCt is the difference of cycle threshold of the target gene with respect to the cycle threshold of the housekeeping gene (*gmk*). The oligonucleotides used for the RT-qPCR (Supplementary Table S1) are extremely selective, both when used on bacterial RNA (more than 99% specificity in amplification of the target gene) and on RNA from plants (no unspecific amplification).

### Metal analysis by ICP-MS

The content of Cd and Zn in *A. thaliana* was determined using an inductively coupled plasma mass spectrometer (ICP-MS, model 820-MS; Bruker, Bremen, Germany) equipped with a collision-reaction interface (CRI) and glass nebulizer (0.4 ml min^−1^). The external standard calibration curve was performed for both metals by serially diluting multielement standard solution (VWR International, Milan, Italy). Single standard solutions of In (at 0.010 mg L^−1^; Merck KGaA, Darmstadt, Germany) and Y (at 0.005 mg L^−1^; Panreac Química, Barcelona, Spain) were used as internal standards. Further details about the used instrumental conditions are already reported (Astolfi et al., 2020, 2021). For each treatment (three replicates per treatment), *A. thaliana* seedlings were pooled and washed once with 1 mM EDTA to remove divalent cations on plant surfaces, rinsed three times with ddH_2_O, and then dried at 60°C for 48 h on an electric stove. Subsequently, approximately 4 mg of seedling samples were accurately weighed using an analytical balance (sensitivity, 0.1 mg; Europe 60; Gibertini Elettronica, Milan, Italy) and placed in graduated polypropylene tubes (Artiglass s. r.l., Due Carrare, PD, Italy). Then, 0.2 ml HNO_3_ (67%, suprapure; Carlo Erba Reagents, Milan, Italy) and 0.1 ml H_2_O_2_ (30%, suprapure; Merck KgaA, Darmstadt, Germany) were added, and the samples were digested in open tubes heated in a water bath (WB12; Argo Lab, Modena, Italy) with electronic temperature control at 95°C (temperature accuracy, ± 0.2°C). According to previous studies (Astolfi et al., 2020), the digestion was completed in 30 min, as indicated by the appearance of a colorless solution. Finally, the digests were left to cool, diluted to 5 ml with deionized water (18.3 MΩ cm resistivity) obtained from an Arioso (Human Corporation, Seoul, Korea) Power I RO-UP Scholar UV deionizer system, and filtered (0.45 μm pore size, GVS Filter Technology, Indianapolis, IN, United States) before ICP-MS analysis. The blanks (3% HNO_3_) were treated as samples together with each series of digested samples to trace possible contamination of the samples and subtract the background signal of the reagents. The method detection and quantification limits (MDL and MQL, respectively) were 0.001 and 0.004 mg kg^−1^ for Cd, and 0.4 and 1 mg kg^−1^ for Zn, respectively.

### Statistical analyses

Statistical analyses were performed using GraphPad Prism v.8.3.1 software. Either Student’s *t*-test or Two-way ANOVA with Tukey’s multiple comparison test was been performed as specified in the figure captions.

## Results

### *Salmonella* Typhimurium ATCC14028s enters and multiplies into *Arabidopsis thaliana* seedlings

**Figure 1:**
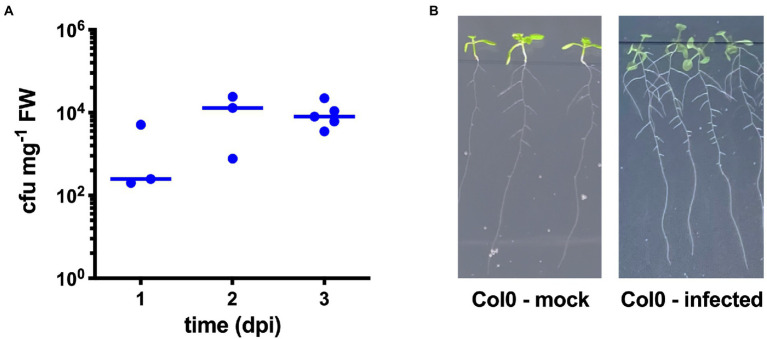
*Salmonella* Typhimurium ATCC14028 (STM) infection of *Arabidopsis thaliana* shoots. **(A)** Enumeration of STM recovered from Col-0 shoots at different time points, each symbol corresponding to a single shoot. Horizontal lines indicate median values. The diagram shows a representative experiment of at least three independent assays. **(B)** Representative pictures of Col-0 seedlings at 3 dpi showing an average increase of lateral roots.

We first checked the ability of STM wild-type strain to colonize *A. thaliana* Col-0 plants. For this purpose, seedlings grown on 1/2 MS were inoculated with STM, and the shoots were harvested at different time points (1–3 dpi), surface-sterilized, and homogenized to evaluate bacterial content. As shown in Figure 1A, STM was always recovered from the plant homogenates, observing an increase of bacterial content at 2 and 3 dpi. We noticed a better homogeneity in the counts of the different samples at 3 dpi, so we decided to use this time point for further inoculation experiments. Overall, according to our data and as already described for other *Salmonella* strains (Schikora et al., 2008), STM can invade *A. thaliana*, confirming the validity and reliability of this model for further experiments. We did not find any evident pathologic alteration in inoculated seedlings, and we observed an average increase in lateral roots number following STM colonization (Figure 1B) as previously reported (Cox et al., 2018).

### Both the Zn high-affinity uptake and the Zn detoxification systems are expressed in STM recovered from *Arabidopsis thaliana*

**Figure 2:**
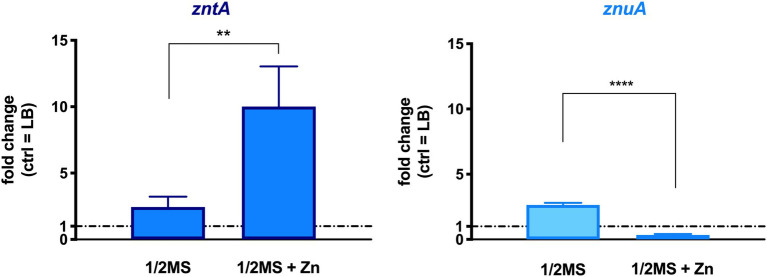
Transcription levels of genes of STM recovered from *Arabidopsis thaliana* infected shoots. RT-qPCR was performed on RNA extracted from plants homogenates, and fold changes were calculated with respect to the gene transcription level from LB-grown STM. Statistical significances were calculated by the Student’s *t*-test (****p < 0.0001; **p < 0.01). The graph is a representative experiment of a two independent assays.

The role of STM Zn homeostasis in plant colonization was first evaluated by checking the transcription levels of *zntA* and *znuA* genes, involved, respectively, in the export of excess Zn and in its uptake under conditions of low Zn availability. Col-0 seedlings, grown in 1/2 MS and in the same medium supplemented with a 10-fold Zn concentration (1/2 MS + Zn), were inoculated with STM, and the shoots were harvested at 3 dpi. RT-qPCR analyses were performed on total RNA extracted from plant homogenates, using, as a control, RNA extracted from STM grown in LB medium. The latter is a condition where ZntA is not required for bacterial growth and ZnuA is almost undetectable (Ammendola et al., 2014). As shown in Figure 2, the induction of *zntA* significantly increases when plants were grown in a Zn-enriched medium, even if it is detectable also in bacteria recovered from plants grown in a medium containing a basal amount of Zn. Intriguingly, also *znuA* was induced in Zn-basal condition. On the contrary, when the medium was supplemented with Zn, *znuA* was significantly repressed (Figure 2).

### ZnuA does not significantly contribute to STM persistence in *Arabidopsis thaliana* shoots

**Figure 3:**
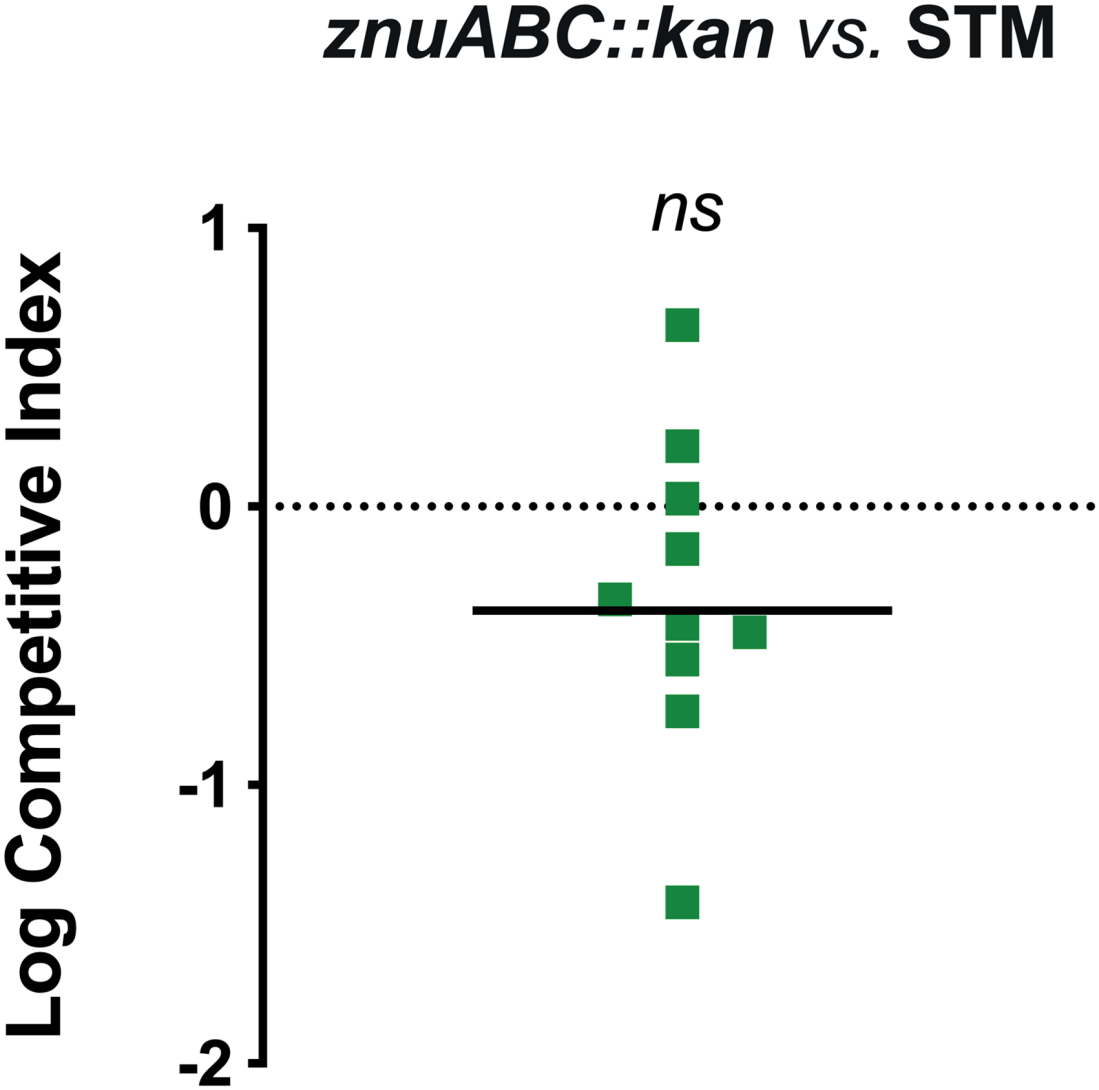
Competition assay in Col-0 between *znuABC::kan* and STM. The seedlings were inoculated with a mixed inoculum of *znuABC::kan* (strain A) and STM (strain B), in a 1:1 ratio. At 3 dpi, the ratio of the two strains was enumerated in shoots homogenates and the competitive index was calculated as described in the section Materials and methods. Statistical significance was obtained by the Student’s *t*-test (*ns*, not significant). A horizontal straight line indicates the median CI value. The graph is representative of two independent experiments.

The eventual contribution of the ZnuABC high-affinity uptake system to STM-plant colonization was analyzed in a competition assay between STM and an isogenic mutant carrying a deletion in the *znuABC* region. This deletion abrogates the functionality of the transporter, severely impairing STM growth in a Zn-restricted environment (Ammendola et al., 2007). As represented in Figure 3, the median CI value calculated over all the analyzed shoots suggests a slight competitive advantage of STM over the *znuABC* mutant. However, we have found significant heterogeneity in the outcome of the competitions. The differences between the input and the output ratios were not statistically significant, leading us to conclude that Zn import through ZnuABC has no critical role in STM-plant interaction.

### ZntA is required for STM colonization of *Arabidopsis thaliana* related to plant Zn/Cd content

To correlate the ability of *Salmonella* to colonize plant tissues to the Zn availability in the plant growth medium and thus to the seedling Zn content, we carried out inoculation experiments with the *A. thaliana* Col-0 and *hma2/hma4* double mutant line, the latter being characterized by the impairment in the Zn and Cd root-to-shoot transport for the absence of the two Divalent Heavy Metal-Transporting P_IB_-type ATPases, HMA2 and HMA4, mainly involved in this process (Hussain et al., 2004). ICP-MS analyses were performed on the shoots of 10-day-old seedlings grown in 1/2 MS and 1/2 MS + Zn.

**Figure 4:**
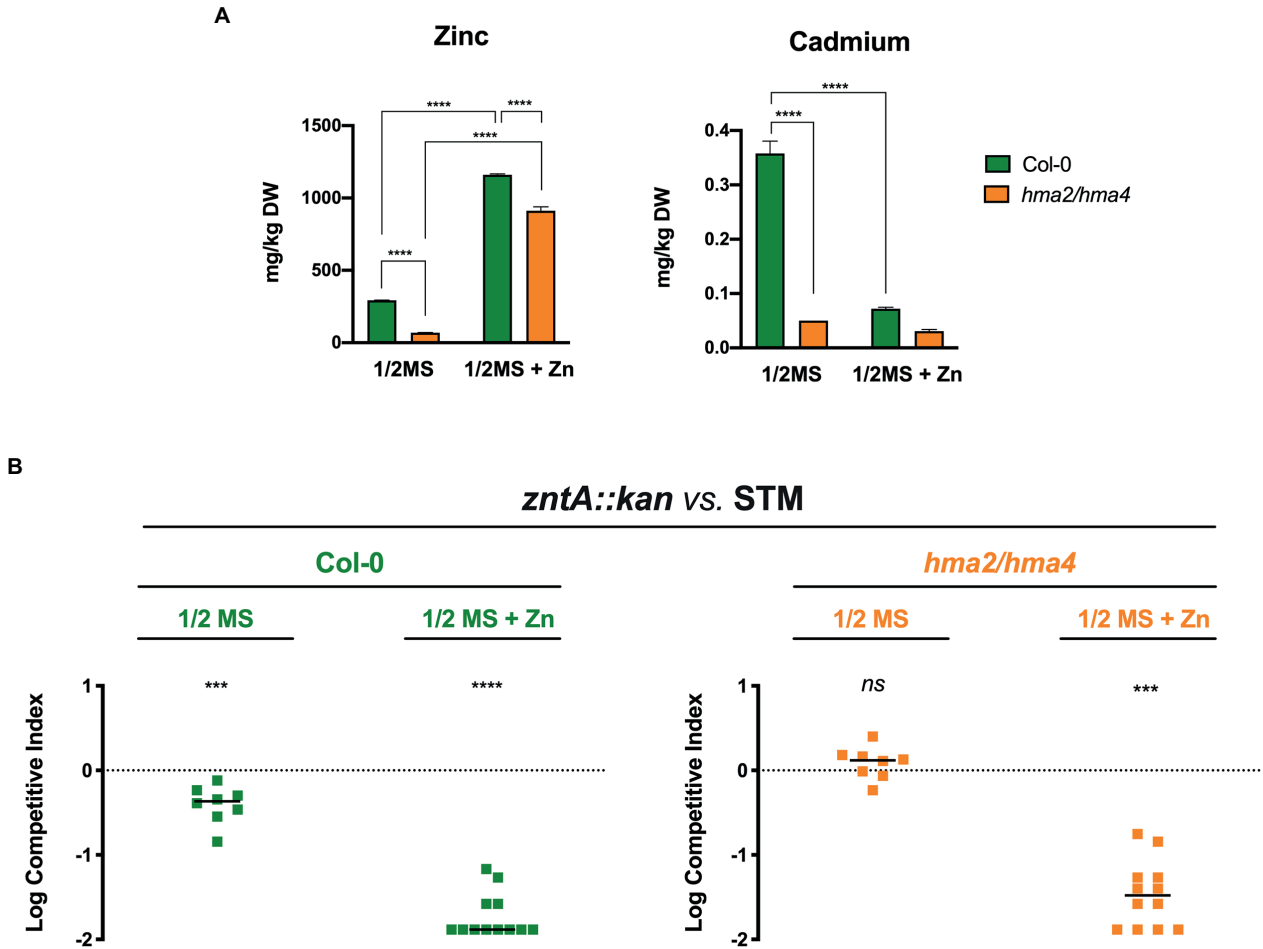
*Salmonella* colonization of *Arabidopsis thaliana* related to Zn and Cd shoots content. **(A)** Inductively coupled plasma mass spectrometer (ICP-MS) analyses of Col-0 and *hma2/hma4* shoots, grown in 1/2MS or 1/2MS + Zn. Statistical differences were calculated by Two-way ANOVA and Sidak’s multiple comparison test (****p < 0.0001). **(B)** Competition assays in Col-0 and *hma2/hma4* between *zntA::kan* (strain A) and STM (strain B). Competitive indexes were calculated as described in section “Materials and methods,” and the statistical significance was determined by the Student’s *t*-test (***p < 0.005; ****p < 0.0001; and ns, not significant). The diagram shows a representative experiment of two independent assays.

The results (Figure 4A) confirmed that Col-0 and *hma2/hma4* plants grown in 1/2 MS contain significantly different Zn levels in the shoots. As expected, the *hma2/hma4* mutant line has more than 4-fold less Zn with respect to Col-0 (69 vs. 293 mg kg^−1^ DW), confirming the importance of HMA2 and HMA4 for a proper Zn translocation to the aerial part of the plant. Zn content in the shoots of both plant lines grown in the Zn supplemented medium (1/2MS + Zn) sharply increased, even if in the *hma2/hma4* mutant it was still lower than that in Col-0. According to the role of these pumps also in Cd transport, the *hma2/hma4* mutant accumulates much less Cd in the shoots compared to Col-0. Interestingly, in the 1/2MS + Zn conditions, Cd content in Col-0 decreases to levels comparable to those detected in *hma2/hma4*, suggesting a metal preference towards Zn of the HMA2 and HMA4 pumps.

Given these results, we performed *Salmonella* competition assays between STM and *zntA::kan* for the colonization of Col-0 or *hma2/hma4* line, grown under different Zn availability. As shown in Figure 4B, we have found that the ability of STM to outcompete the *zntA::kan* mutant is dependent on the Zn/Cd content in the shoots. The competitive advantage of STM is always significant in Col-0 shoots, both when the Zn provided to the seedlings is relatively low (1/2MS) and even more if Zn availability is increased (1/2MS + Zn). In contrast, the two strains have a comparable ability to colonize the shoots of plants that contain a much lower amount of Zn, i.e., the *hma2/hma4* line grown in 1/2 MS. These results underline the role of ZntA in dealing with Zn/Cd levels in plant tissues.

### ZitB makes a substantial contribution to metal detoxification in Zn-rich environments

It has been suggested that the cation diffusor facilitator ZitB plays an auxiliary role in Zn export during *Salmonella* infection of Nramp^+^ macrophages (Huang et al., 2018). To verify whether it could contribute to *Salmonella* persistence in plant tissues, we have constructed two strains carrying a *zitB::cam* mutation and a double *zntA::kan zitB::cam* mutation. The sensitivity toward Zn of these two strains was compared to that of STM and *zntA::kan* mutant by measuring their optical densities in VBMM supplemented with different amounts of metal (Supplementary Figure S1).

**Figure 5:**
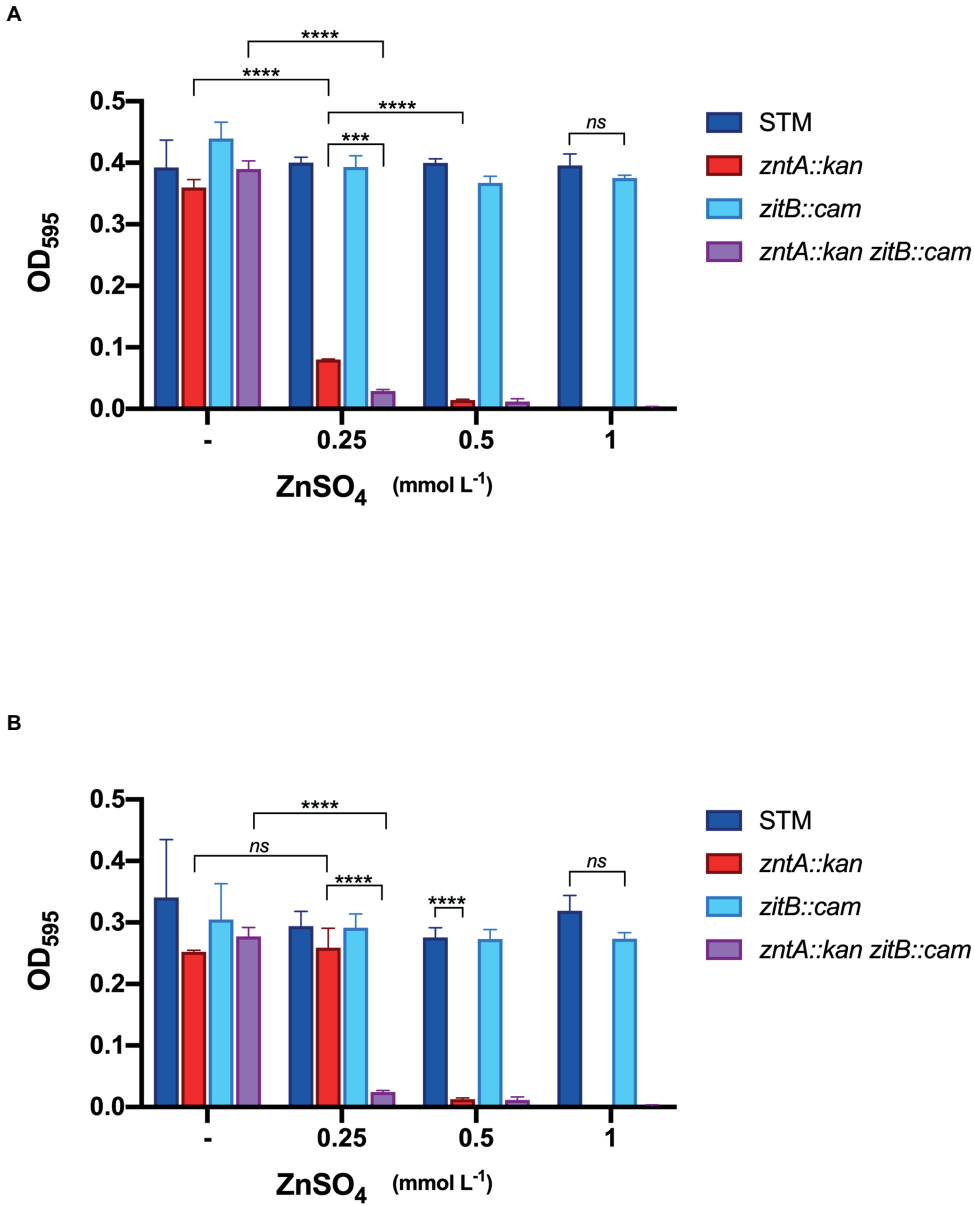
*In vitro* sensitivity of STM and mutants to Zn. *Salmonella* strains were grown in Vogel Bonner Minimal Medium (VBMM) supplemented with increasing concentrations of ZnSO_4_ as indicated. Optical densities at 595 nm were recorded after 9 h **(A)** and 18 h **(B)**. Each bar represents the mean value, and statistical significances are calculated by Two-way ANOVA and Tukey’s multiple comparison test (***p < 0.005; ^****^p < 0.0001; and ns, not significant). Only significative differences between bars discussed in the text are shown; the overall statistical results are reported in Supplementary Table S2. The graphs are representative experiments of three independent assays.

After 9 h (Figure 5A), all the strains cultured in standard VBMM reached the stationary phase of growth while, in the presence of even the lowest Zn supplement, the *zntA::kan* strain showed a significant growth impairment, confirming the primary role of this exporter in detoxification of Zn excess. The absence of ZitB alone did not cause any growth difference with wild type STM (even at the highest Zn concentration). Interestingly, a more pronounced growth reduction was evidenced in the Δ*zntAzitB* double mutant than in the *zntA::kan* strain. The additive effect of the two deletions on the impairment of the metal detoxification mechanism was more evident after prolonged growth of the strains (Figure 5B). In particular, in the presence of 0.25 mmol L^−1^ ZnSO_4_, the double mutant still failed to grow, the *zntA* mutant was able to adapt to metal supplementation and reached the same densities of STM, while the double mutant still failed to growth. These observations suggest that ZitB has a role in adapting *Salmonella* to Zn-rich environments, which becomes crucial if the primary detoxification system is impaired.

### ZitB alleviates metal stress conditions in STM-plant colonization

**Figure 6:**
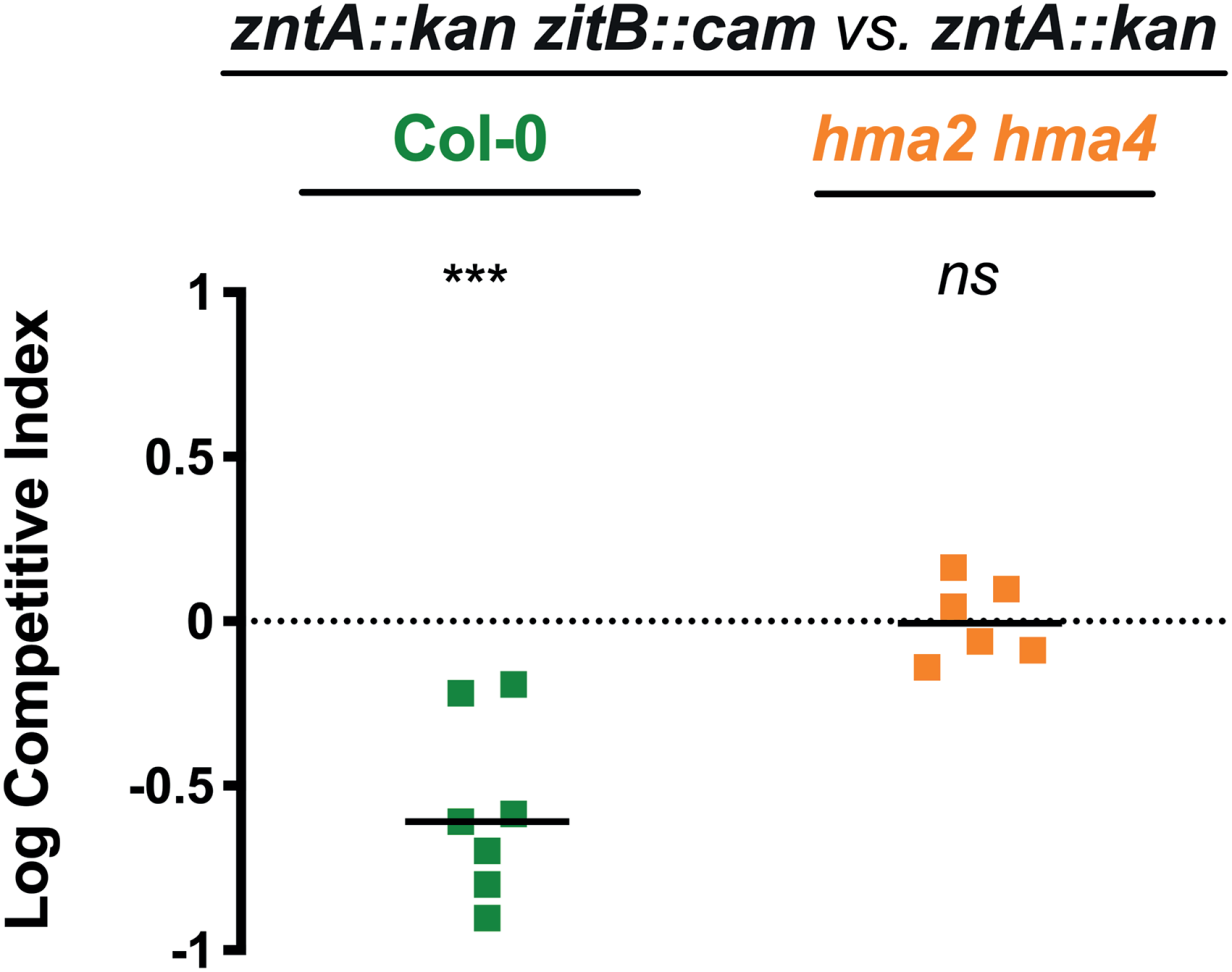
The lack of ZitB influences *Salmonella* persistence in *Arabidopsis thaliana* shoots. Competition assays in Col-0 and *hma2/hma4* between *zntA::kan zitB::cam* (strain A) and *zntA::kan* (strain B). Competitive indexes were calculated as described in section “Materials and methods,” and the statistical significance was determined by the Student’s *t*-test (***p < 0.005; ns, not significant). The diagram shows a representative experiment of two independent assays.

In the light of the above findings, we wondered if ZitB could have a role in STM persistence in plants. To disclose this possibility, we performed competition assays between the double mutant, *zntA::kan zitB::cam*, and the single mutant *zntA*. Inoculation experiments were performed both on Col-0 and *hma2/hma4* plants, grown in 1/2 MS, a condition where the presence of a functional ZntA exporter is advantageous to bacterial persistence into Col-0 but disposable in *hma2/hma4* colonization. As shown in Figure 6, the simultaneous loss of the two Zn efflux systems causes a significant competitive disadvantage for the *zntA::kan zitB::cam* strain with respect to the lack of ZntA alone. This difference is only detectable in Col-0, while in the *hma2/hma4* line, the two strains display a similar ability to colonize the shoots. These data further suggest that the ability of STM to colonize plant tissue can also rely on functional multiple metal detoxification systems and correlates to the Zn status of the plant.

## Discussion

Plant contamination by *Salmonella enterica* can occur through invasion and colonization of the plant tissues, similar to that of endophytic plant pathogens. The interaction between *Salmonella* and different plant species, including the edible ones, has been investigated in the last decades, focusing both on the plant response mechanisms and on the bacterial pathways required for invasion and survival. Some studies pointed out that most plant response mechanisms are conserved, and that, among bacterial virulence factors required for successful colonization, both flagellin and T3SSs have a role in plant infection and in the translocation of effector proteins that enhance bacterial survival in plant tissues (Zarkani and Schikora, 2021). To date, it is not clear if plants respond to pathogen invasion by modulating metal concentration in their tissues. It has been hypothesized that hyperaccumulator plants exploit high zinc concentration to counteract pathogens, either by direct metal toxicity or by enhancing Zn-triggered plant defenses (Fones et al., 2019). However, in non-accumulator plants, few studies have investigated the correlation between plant metal content and pathogen survival in plant tissues.

The importance of metal detoxification systems in the virulence of bacterial pathogens has been mainly analyzed using animal models of infections and it is still controversial. In *Yersinia pestis*, ZntA plays a role in resistance to Zn toxicity *in vitro*, but a mutant lacking this exporter retains full virulence in mice models and survival in macrophages (Botella et al., 2011; Kapetanovic et al., 2016; Bobrov et al., 2017). A *S.* Typhimurium strain lacking both ZntA and ZitB is impaired in Nramp-1 positive mice, while its survival in Nramp-1 negative macrophages and amoebae models is not affected, supporting the role of Nramp-1 phagosomal pump in an intoxication mechanism involving zinc (Huang et al., 2018). In plant models, a study on *Xylella fastidiosa* has shown that the alteration of bacterial zinc homeostasis, including the impairment of detoxification systems, causes a reduced ability to trigger symptoms in *Nicotiana tabacum*. This suggests that host Zn levels can limit the growth of *X. fastidiosa* in plants and interfere with its virulence (Navarrete and De La Fuente, 2015).

In the present research, we have chosen *A. thaliana* as a model system to investigate the role of ZntA and ZitB in the ability of *Salmonella* to colonize plant tissues. The capacity of *Salmonella* to actively invade and proliferate in the aerial portion of *Arabidopsis* seedlings was already described for other plants, likely exploiting the stomata as a route of entry (Schikora et al., 2008; Kroupitski et al., 2009). Even in our experiments *S.* Typhimurium 14,028, a virulent strain in animal models of infection, proved to be able to invade *A. thaliana* shoots and grow in plant tissues (Figure 1A). This finding was not obvious, as the colonization ability of *S.* Typhimurium in plants has been shown to be strain-dependent. Interestingly at 3 dpi, an increased number of lateral roots were detected (Figure 1B). This phenotype has been already observed in *Salmonella* infected *Medicago truncatula* seedlings and has been related to the production by the pathogen of indole-3-acetic acid (Cox et al., 2018), which is the main auxin that regulates plant growth and is known to promote lateral root formation (Muday and Haworth, 1994).

The role of the main *Salmonella* effectors of zinc homeostasis was analyzed in our model, comparing the transcription levels of *znuA* and *zntA* genes in bacteria colonizing plants grown in different Zn availability conditions, i.e., a Zn-basal or a Zn-enriched medium (Figure 2). The slight induction of both genes under Zn-basal conditions may appear contradictory; however, we have already observed a similar simultaneous expression of ZntA and ZnuA in bacteria facing a cadmium-stress condition that deregulates the Zn homeostatic systems (Ammendola et al., 2014). Therefore, we may hypothesize that, in plant tissues, *Salmonella* senses a stress condition involving toxic metal accumulation. The expression of *znuA* is abolished by a 10-fold increased concentration of Zn in the plant growth medium. In this latter condition, in fact, *Salmonella* clearly responds to a Zn excess by upregulating the exporter ZntA.

As expected, the plant Zn content was indeed modulated by the amount of Zn in the growth medium (Figure 4): its concentration in the shoots sharply increases when plants grow in a Zn-rich medium, while, at Zn-basal levels, the accumulation of the metal in the shoots is strongly dependent on the presence of the P1B-ATPases Heavy Metal ATPase2 and Heavy Metal ATPase4 (HMA2 and HMA4). These metal transporters are localized on the plasma membrane of vascular cells and are the main root-to-shoot Zn translocation system (Hussain et al., 2004). Interestingly, we have found that Cd content can be influenced by Zn availability, as its concentration significantly decreases under Zn-rich plant growth conditions. Moreover, we have observed that *hma2/hma4* shoots have a reduced Cd content compared to Col-0 (Figure 4A), suggesting that Cd translocation root-to-shoot is mainly mediated by HMA2 and/or HMA4. The role of these transporters in Cd mobilization has been already suggested, only indirectly, by heterologous expression in yeast, where Cd resistance was increased upon HMA4 overexpression (Mills et al., 2003). Our data suggest that Cd content of the Col-0 line grown in the Zn-basal condition is likely sensed by colonizing bacteria as a metal-stress condition, causing deregulation of Zn homeostasis mechanism (Figure 2).

To correlate the regulation of *znuA* and *zntA* with the ability of *Salmonella* to persist in plant tissues, we performed competition assays between the strains with an impaired Zn homeostasis, either the *znuABC* or the *zntA* deleted mutants, and STM. The advantage of competition assays, which have been already used in animal infection studies, is that of minimizing effects due to uncontrolled experimental variability and thus directly comparing the fitness of two different strains in the same niche at the same time. This could be particularly useful in inoculation experiments of plants, where both *Salmonella* growth conditions and plant status have shown to greatly influence the outcome of the infection (Kroupitski et al., 2009; Oblessuc et al., 2020).

The competition assays showed that the absence of ZnuABC does not confer a significant impairment to *Salmonella* in plant colonization (Figure 3), meaning that in this environment Zn availability for bacterial survival is not a limiting factor and supporting our hypothesis that the slight induction of *znuA* gene represents a “side effect” of a Cd-stress response. On the contrary, the impairment of the Zn/Cd detoxification system significantly impacts *Salmonella* fitness in plant tissues, even in Zn-basal conditions. This effect was greatly enhanced in plants grown in a Zn-enriched medium, where the *zntA* mutant was strongly outcompeted by STM, due to the higher Zn accumulation in the shoots (Figure 4B, left panel).

In the absence of HMA2 and HMA4, the *Salmonella* Zn/Cd detoxification system is not critical for bacterial persistence in the shoots. In fact, we have found no significant differences between the *zntA* mutant and STM in *hma2/hma4*, indicating that the lower amount of zinc in the shoots does not require the activity of ZntA. On the contrary, when the mutant plant line was grown in a Zn-rich medium, the increase of Zn in the shoots confers a strong competitive advantage to STM over the *zntA* mutant (Figure 4B, right panel).

HMA2 and HMA4 transporters are overexpressed in hyperaccumulator plants, such as *A. halleri*. Compared to non-accumulator plants, characterized by a higher concentration of metals at the level of the root system, hyperaccumulator plants have a higher concentration of metals in the leaves. Besides the role of these transporters in plant adaptation to soils with high metal concentrations, it has also been suggested that the ability to accumulate high levels of metals in the shoots could be a defense mechanism against herbivorous and pathogens (Hörger et al., 2013). Recently, a role of HMA2 and HMA4 in the defense of *A. thaliana* against the pathogenic fungus *Plectosphaerella cucumerina* has been suggested, hypothesizing a mechanism of Zn-mediated immunity based on the active accumulation of Zn at the sites of infection through the upregulation of the plant transporters (Escudero et al., 2022).

We have found that the ability of *Salmonella* to persist in plant tissues, besides the expression of the main Zn/Cd detoxification system, was dependent on the presence of ZitB. This Cation Diffusor Facilitator family member was first identified in *E. coli* as a metal exporter induced by *in vitro* ZnCl_2_ exposure, whose deletion caused hypersensitivity to Zn only when combined with the lack of ZntA (Grass et al., 2005). In *S.* Typhimurium strain 4/74, ZitB was described to have a role in Nramp+ macrophages and mice infections, together with ZntA (Huang et al., 2018). Differently from *E. coli*, *S.* Typhimurium ZitB appears to be constitutively regulated, and it is required for the resistance to intracellular Zn increase following nitrosative stress, together with ZntA (Frawley et al., 2018). More recently ZitB was also shown to be constitutively expressed in *Klebsiella pneumoniae* as a part of the Zn and Cd detoxification system (Maunders et al., 2022).

In the attempt to analyze the contribution of ZitB to plant colonization, we first characterized the *in vitro* growth phenotype of strains carrying a deletion in *zitB*, alone or in combination with a *zntA* deletion, and compared their sensitivity toward Zn with that of STM and the single *zntA* deletion mutant (Figure 5). In agreement with previous findings, our result pointed out that, although ZntA is the main effector of Zn resistance, a contribution of ZitB could be noticed at the lower Zn concentration tested (ZnSO_4_ 0.25 mmol L^−1^, Figure 5A). Interestingly, at the same Zn concentration and after a prolonged growth (18 h, Figure 5B), we have noticed the recovery of the *zntA* mutant, which reached similar growth densities as STM and *zitB* mutant strains. However, in the same condition, the double *zntAzitB* mutant still failed to grow, indicating an additive role of ZitB in the ZntA-mediated Zn detoxification mechanism.

The results of competition assays between *zntA* and *zntAzitB* mutant strains further supported this hypothesis and confirmed the role of ZitB also *in vivo*, during plant colonization. The Zn levels found in the shoots of Col-0 plants grown in Zn-basal medium are sufficient to confer a competitive advantage of *zntA::kan* vs. *zntA::kan zitB::cam*, which is abolished in the *hma2/hma4* colonization (Figure 6). As previously shown, in this latter condition the Zn content is significantly lower with respect to Col-0 shoots (Figure 4A). Even if ZitB itself has apparently a lower impact in detoxifying the cell from the excess of metals compared to ZntA, these data highlight the importance of a complete and functional detoxification system for the survival and proliferation of *Salmonella* in plants.

Overall, our data suggest that, in order to colonize plant tissues, *Salmonella* takes advantage of its ability to export excess metal through multiple efflux mechanisms. Depending on the medium composition, even non-accumulator plants can increase Zn content in their tissues to levels that are sensed by colonizing bacteria as toxic. For this reason, the absence of a functional metal detoxification system can impair *Salmonella* persistence in the shoots. We have also shown that the *Salmonella* growth disadvantage related to zinc detoxification impairment can be abrogated if the plant is unable to efficiently translocate zinc to the shoots. Further studies are needed to understand if there is a differential accumulation of zinc as a response of plants to the invasion of *Salmonella*, for example by activating plant-specific transporters and if the mechanism of Zn accumulation could also be found in other plant species, with a particular interest in edible ones. Plant contamination by *S.* Typhimurium represents an important threat to public health and an economic problem for the food industry. For this reason, understanding the molecular mechanisms that drive the interaction between *Salmonella* and its plant host could also be useful for the improvement of agricultural practices in order to limit the colonization of pathogens. It is currently known that some edible crops, such as *Brassicaceae* and *Spinacia oleracea*, can accumulate Zn in leaves up to concentrations of 300 mg Kg^−1^ without showing symptoms of toxicity (Mishra et al., 2020). Even though Zn bioaccumulation in these plants is an order of magnitude less than detected in hyperaccumulators, it could be exploited by the plant as defense mechanism to counteract pathogen colonization.

For these reasons, biofortification with zinc, besides improving the crop nutritional quality, could also represent a promising strategy for protection against plant colonization by bacterial pathogens (Stanton et al., 2022).

## Data availability statement

The raw data supporting the conclusions of this article will be made available by the authors, without undue reservation.

## Author contributions

SA, SV, and AB: conceptualization. SA, SV, and MA: investigation. SA, SV, MA, and AB: writing—original draft preparation and writing—review and editing. All authors contributed to the article and approved the submitted version.

## Funding

This work was supported by ZIPLANT—Progetto di Ricerca Scientifica di Ateneo 2021, University of Rome Tor Vergata.

## Conflict of interest

The authors declare that the research was conducted in the absence of any commercial or financial relationships that could be construed as a potential conflict of interest.

## Publisher’s note

All claims expressed in this article are solely those of the authors and do not necessarily represent those of their affiliated organizations, or those of the publisher, the editors and the reviewers. Any product that may be evaluated in this article, or claim that may be made by its manufacturer, is not guaranteed or endorsed by the publisher.

## References

[ref1] Ammendola S. Cerasi M. Battistoni A. (2014). Deregulation of transition metals homeostasis is a key feature of cadmium toxicity in salmonella. Biometals 27, 703–714. doi: 10.1007/s10534-014-9763-2, PMID: 24970347

[ref2] Ammendola S. D’Amico Y. Chirullo B. Drumo R. Civardelli D. Pasquali P.. (2016). Zinc is required to ensure the expression of flagella and the ability to form biofilms in *Salmonella enterica* sv *Typhimurium*. Metallomics 8, 1131–1140. doi: 10.1039/C6MT00108D, PMID: 27730246

[ref3] Ammendola S. Pasquali P. Pistoia C. Petrucci P. Petrarca P. Rotilio G.. (2007). High-affinity Zn2+ uptake system ZnuABC is required for bacterial zinc homeostasis in intracellular environments and contributes to the virulence of *Salmonella enterica*. Infect. Immun. 75, 5867–5876. doi: 10.1128/IAI.00559-07, PMID: 17923515 PMC2168356

[ref4] Astolfi M. L. Conti M. E. Marconi E. Massimi L. Canepari S. (2020). Effectiveness of different sample treatments for the elemental characterization of bees and beehive products. Molecules 25:4263. doi: 10.3390/MOLECULES25184263, PMID: 32957599 PMC7570605

[ref5] Astolfi M. L. Marconi E. Vitiello G. Massimi L. (2021). An optimized method for sample preparation and elemental analysis of extra-virgin olive oil by inductively coupled plasma mass spectrometry. Food Chem. 360, 130027. doi: 10.1016/J.FOODCHEM.2021.130027, PMID: 34029926

[ref6] Barwinska-Sendra A. Waldron K. J. (2017). The role of intermetal competition and Mis-Metalation in metal toxicity. Adv. Microb. Physiol. 70, 315–379. doi: 10.1016/bs.ampbs.2017.01.003, PMID: 28528650

[ref7] Berens M. L. Berry H. M. Mine A. Argueso C. T. Tsuda K. (2017). Evolution of hormone signaling networks in plant defense. Annu. Rev. Phytopathol. 55, 401–425. doi: 10.1146/ANNUREV-PHYTO-080516-035544, PMID: 28645231

[ref8] Birkenbihl R. P. Liu S. Somssich I. E. (2017). Transcriptional events defining plant immune responses. Curr. Opin. Plant Biol. 38, 1–9. doi: 10.1016/J.PBI.2017.04.004, PMID: 28458046

[ref9] Bobrov A. G. Kirillina O. Fosso M. Y. Fetherston J. D. Miller M. C. Vancleave T. T.. (2017). Zinc transporters YbtX and ZnuABC are required for the virulence of: Yersinia pestis in bubonic and pneumonic plague in mice. Metallomics 9, 757–772. doi: 10.1039/c7mt00126f, PMID: 28540946 PMC5532734

[ref10] Botella H. Peyron P. Levillain F. Poincloux R. Poquet Y. Brandli I.. (2011). Mycobacterial p(1)-type ATPases mediate resistance to zinc poisoning in human macrophages. Cell Host Microbe 10, 248–259. doi: 10.1016/J.CHOM.2011.08.006, PMID: 21925112 PMC3221041

[ref11] Cabot C. Martos S. Llugany M. Gallego B. Tolrà R. Poschenrieder C. (2019). A role for zinc in plant defense against pathogens and herbivores. Front. Plant Sci. 10:1171. doi: 10.3389/fpls.2019.01171, PMID: 31649687 PMC6794951

[ref12] Cerasi M. Ammendola S. Battistoni A. (2013). Competition for zinc binding in the host-pathogen interaction. Front. Cell. Infect. Microbiol. 3:108. doi: 10.3389/fcimb.2013.00108, PMID: 24400228 PMC3872050

[ref13] Chandrangsu P. Rensing C. Helmann J. D. (2017). Metal homeostasis and resistance in bacteria. Nat. Rev. Microbiol. 15, 338–350. doi: 10.1038/NRMICRO.2017.15, PMID: 28344348 PMC5963929

[ref14] Cox C. E. Brandl M. T. de Moraes M. H. Gunasekera S. Teplitski M. (2018). Production of the plant hormone auxin by salmonella and its role in the interactions with plants and animals. Front. Microbiol. 8:2668. doi: 10.3389/fmicb.2017.02668, PMID: 29375530 PMC5770404

[ref15] Datsenko K. Wanner B. (2000). One-step inactivation of chromosomal genes in Escherichia coli K-12 using PCR products. Proc. Natl. Acad. Sci. 97, 6640–6645. doi: 10.1073/pnas.120163297, PMID: 10829079 PMC18686

[ref16] Djoko K. Y. Ong C. L. Walker M. J. McEwan A. G. (2015). The role of copper and zinc toxicity in innate immune defense against bacterial pathogens. J. Biol. Chem. 290, 18954–18961. doi: 10.1074/JBC.R115.647099, PMID: 26055706 PMC4521016

[ref17] Escudero V. Ferreira Sánchez D. Abreu I. Sopeña-Torres S. Makarovsky-Saavedra N. Bernal M.. (2022). Arabidopsis thaliana Zn2+−efflux ATPases HMA2 and HMA4 are required for resistance to the necrotrophic fungus *Plectosphaerella cucumerina* BMM. J. Exp. Bot. 73, 339–350. doi: 10.1093/jxb/erab400, PMID: 34463334

[ref18] Fiorillo A. Mattei M. Aducci P. Visconti S. Camoni L. (2020). The salt tolerance related protein (STRP) mediates cold stress responses and Abscisic acid Signalling in *Arabidopsis thaliana*. Front. Plant Sci. 11:1251. doi: 10.3389/FPLS.2020.01251, PMID: 32903596 PMC7438554

[ref19] Fitzsimmons L. Liu L. Porwollik S. Chakraborty S. Desai P. Tapscott T.. (2018). Zinc-dependent substrate-level phosphorylation powers salmonella growth under nitrosative stress of the innate host response. PLoS Pathog. 14, e1007388. doi: 10.1371/JOURNAL.PPAT.1007388, PMID: 30365536 PMC6221366

[ref20] Fones H. N. Preston G. M. Smith J. A. C. (2019). Variation in defence strategies in the metal hyperaccumulator plant Noccaea caerulescens is indicative of synergies and trade-offs between forms of defence. R. Soc. Open Sci. 6:172418. doi: 10.1098/rsos.172418, PMID: 30800336 PMC6366173

[ref21] Frawley E. R. Karlinsey J. E. Singhal A. Libby S. J. Doulias P. T. Ischiropoulos H.. (2018). Nitric oxide disrupts zinc homeostasis in salmonella enterica serovar typhimurium. mBio 9, 1–23. doi: 10.1128/mBio.01040-18, PMID: 30108168 PMC6094482

[ref22] Grass G. Franke S. Taudte N. Nies D. H. Kucharski L. M. Maguire M. E.. (2005). The metal permease ZupT from Escherichia coli is a transporter with a broad substrate spectrum. J. Bacteriol. 187, 1604–1611. doi: 10.1128/JB.187.5.1604-1611.2005, PMID: 15716430 PMC1064025

[ref23] Hörger A. C. Fones H. N. Preston G. M. (2013). The current status of the elemental defense hypothesis in relation to pathogens. Front. Plant Sci. 4:395. doi: 10.3389/FPLS.2013.00395, PMID: 24137169 PMC3797420

[ref24] Huang K. Wang D. Frederiksen R. F. Rensing C. Olsen J. E. Fresno A. H. (2018). Investigation of the role of genes encoding zinc exporters zntA, zitB, and fieF during salmonella Typhimurium infection. Front. Microbiol. 8:2656. doi: 10.3389/fmicb.2017.02656, PMID: 29375521 PMC5768658

[ref25] Hussain D. Haydon M. J. Wang Y. Wong E. Sherson S. M. Young J.. (2004). P-type ATPase heavy metal transporters with roles in essential zinc homeostasis in arabidopsis. Plant Cell 16, 1327–1339. doi: 10.1105/tpc.020487, PMID: 15100400 PMC423219

[ref26] Kapetanovic R. Bokil N. J. Achard M. E. S. Ong C. L. Y. Peters K. M. Stocks C. J.. (2016). Salmonella employs multiple mechanisms to subvert the TLR-inducible zinc-mediated antimicrobial response of human macrophages. FASEB J. 30, 1901–1912. doi: 10.1096/FJ.201500061, PMID: 26839376

[ref27] Kroupitski Y. Golberg D. Belausov E. Pinto R. Swartzberg D. Granot D.. (2009). Internalization of salmonella enterica in leaves is induced by light and involves chemotaxis and penetration through open stomata. Appl. Environ. Microbiol. 75, 6076–6086. doi: 10.1128/AEM.01084-09, PMID: 19648358 PMC2753090

[ref28] Liu J. Z. Jellbauer S. Poe A. J. Ton V. Pesciaroli M. Kehl-Fie T. E.. (2012). Zinc sequestration by the neutrophil protein calprotectin enhances salmonella growth in the inflamed gut. Cell Host Microbe 11, 227–239. doi: 10.1016/j.chom.2012.01.017, PMID: 22423963 PMC3308348

[ref29] Maunders E. A. Ganio K. Hayes A. J. Neville S. L. Davies M. R. Strugnell R. A.. (2022). The role of ZntA in Klebsiella pneumoniae zinc homeostasis. Microbiol. Spectr. 10, e0177321. doi: 10.1128/SPECTRUM.01773-21, PMID: 35019689 PMC8754117

[ref30] McDevitt C. A. Ogunniyi A. D. Valkov E. Lawrence M. C. Kobe B. McEwan A. G.. (2011). A molecular mechanism for bacterial susceptibility to zinc. PLoS Pathog. 7, e1002357. doi: 10.1371/journal.ppat.1002357, PMID: 22072971 PMC3207923

[ref31] Mills R. F. Krijgert G. C. Baccarini P. J. Hall J. L. Williams L. E. (2003). Functional expression of AtHMA4, a P1B-type ATPase of the Zn/co/cd/Pb subclass. Plant J. 35, 164–176. doi: 10.1046/J.1365-313X.2003.01790.X, PMID: 12848823

[ref32] Mishra B. McDonald L. M. Roy M. Lanzirotti A. Myneni S. C. B. (2020). Uptake and speciation of zinc in edible plants grown in smelter contaminated soils. PLoS One 15, e0226180. doi: 10.1371/JOURNAL.PONE.0226180, PMID: 32302305 PMC7164604

[ref33] Muday G. Haworth P. (1994). Tomato root growth, gravitropism, and lateral development: correlation with auxin transport. Plant Physiol. Biochem. 32, 193–203. 11540612

[ref34] Murdoch C. C. Skaar E.P. (2022). Nutritional immunity: the battle for nutrient metals at the host-pathogen interface. Nat. Rev. Microbiol. doi: 10.1038/s41579-022-00745-6 [Epub ahead of print]. PMC9153222 35641670

[ref35] Navarrete F. De La Fuente L. (2015). Zinc detoxification is required for full virulence and modification of the host leaf ionome by Xylella fastidiosa. Mol. Plant-Microbe Interact. 28, 497–507. doi: 10.1094/MPMI-07-14-0221-R, PMID: 25561271

[ref36] Núñez G. Sakamoto K. Soares M. P. (2018). Innate nutritional immunity. J. Immunol. 201, 11–18. doi: 10.4049/jimmunol.1800325, PMID: 29914937 PMC6028930

[ref37] Oblessuc P. R. Matiolli C. C. Melotto M. (2020). Novel molecular components involved in callose-mediated Arabidopsis defense against salmonella enterica and Escherichia coli O157:H7. BMC Plant Biol. 20, 16–13. doi: 10.1186/s12870-019-2232-x, PMID: 31914927 PMC6950905

[ref38] Pasquali P. Ammendola S. Pistoia C. Petrucci P. Tarantino M. Valente C.. (2008). Attenuated salmonella enterica serovar Typhimurium lacking the ZnuABC transporter confers immune-based protection against challenge infections in mice. Vaccine 26, 3421–3426. doi: 10.1016/j.vaccine.2008.04.036, PMID: 18499306

[ref39] Schikora A. Carreri A. Charpentier E. Hirt H. (2008). The dark side of the salad: salmonella typhimurium overcomes the innate immune response of arabidopsis thaliana and shows an endopathogenic lifestyle. PLoS One 3, e2279. doi: 10.1371/journal.pone.0002279, PMID: 18509467 PMC2386236

[ref40] Sheldon J. R. Skaar E. P. (2019). Metals as phagocyte antimicrobial effectors. Curr. Opin. Immunol. 60, 1–9. doi: 10.1016/J.COI.2019.04.002, PMID: 31063946 PMC6800623

[ref41] Stanton C. Sanders D. Krämer U. Podar D. (2022). Zinc in plants: integrating homeostasis and biofortification. Mol. Plant 15, 65–85. doi: 10.1016/J.MOLP.2021.12.008, PMID: 34952215

[ref42] Wang D. Hosteen O. Fierke C. A. (2012). ZntR-mediated transcription of zntA responds to nanomolar intracellular free zinc. J. Inorg. Biochem. 111, 173–181. doi: 10.1016/J.JINORGBIO.2012.02.008, PMID: 22459916 PMC3408962

[ref43] Zarkani A. A. Schikora A. (2021). Mechanisms adopted by salmonella to colonize plant hosts. Food Microbiol. 99:103833. doi: 10.1016/j.fm.2021.103833, PMID: 34119117

[ref44] Zheng J. Allard S. Reynolds S. Millner P. Arce G. Blodgett R. J.. (2013). Colonization and internalization of salmonella enterica in tomato plants. Appl. Environ. Microbiol. 79, 2494–2502. doi: 10.1128/AEM.03704-12, PMID: 23377940 PMC3623171

