# Supplementary material for: Impairment of the Zn/Cd detoxification systems affects the ability of Salmonella to colonize Arabidopsis thaliana
Source: Front Microbiol. 2022 Aug 22;13:975725. doi: 10.3389/fmicb.2022.975725 (PMC9441889; doi:10.3389/fmicb.2022.975725)

Supplementary Material

# Supplementary Table S1

Oligonucleotides used in this study

| Name | Sequence (5' - 3') | Use |
| --- | --- | --- |
| **K3** | AGCTCACCGTCTTTCATTGC | internal Cam cassette (*zitB::cam* check - forward) |
| **oli-290** | ATAATGCTCGTCGCCTGCTTTTCGCCTTTATCGTCACTGCTGTAGGCTGGAGCTGCTTCG | Forward for Cam cassette amplification on pKD3 (5’ *zitB* homology) |
| **oli-291** | TATTCGTGCATCAGGAAATCCTGGATACGTTCCAGCAGCGCATATGAATATCCTCCTTAG | Reverse for Cam cassette amplification on pKD3 (3’ *zitB* homology) |
| **oli-292** | CGATGTCGCTGGAATATCTG | downstream *zitB* (*zitB::cam* check - reverse) |
| ***zntA* Fw** | GCCTGTTTCTACTGCCATGG | RT-PCR |
| ***zntA* Rev** | ACCGCATTCTCCACTTTTCG | RT-PCR |
| ***znuA* Fw** | caggttaccattgcgcaact | RT-PCR |
| ***znuA* Rev** | cgtatgcgcatgttcatcct | RT-PCR |
| ***gmk* Fw** | CCGCCGTCAAAGATCGAATT | RT-PCR |
| ***gmk Rev*** | ATGGCTCATTTCTGCAACCG | RT-PCR |

# Supplementary Table S2.

Statistical analyses of the OD values, calculated by two-way ANOVA and Tukey's multiple comparison test, relative to data reported in panel A (S2.A) and panel B (S2.B) of figure 5.

**S2.A**

| **Within each column, compare rows (simple effects within columns)** | | | | | | | | |
| --- | --- | --- | --- | --- | --- | --- | --- | --- |
| Tukey's multiple comparisons test | Significant? | | Summary | | | | Adjusted P Value | |
|  |  | |  | | | |  | |
| **STM** |  | |  | | | |  | |
| 0 vs. 0.25 | Yes | | ** | | | | 0,003 | |
| 0 vs. 0.5 | No | | ns | | | | 0,0665 | |
| 0 vs. 1 | Yes | | ** | | | | 0,0018 | |
| 0.25 vs. 0.5 | No | | ns | | | | 0,679 | |
| 0.25 vs. 1 | No | | ns | | | | 0,934 | |
| 0.5 vs. 1 | No | | ns | | | | 0,3933 | |
|  |  | |  | | | |  | |
| ***zntA::kan*** |  | |  | | | |  | |
| 0 vs. 0.25 | Yes | | **** | | | | <0,0001 | |
| 0 vs. 0.5 | Yes | | **** | | | | <0,0001 | |
| 0 vs. 1 | Yes | | **** | | | | <0,0001 | |
| 0.25 vs. 0.5 | Yes | | **** | | | | <0,0001 | |
| 0.25 vs. 1 | Yes | | **** | | | | <0,0001 | |
| 0.5 vs. 1 | No | | ns | | | | 0,9986 | |
|  |  | |  | | | |  | |
| ***zitB::cam*** |  | |  | | | |  | |
| 0 vs. 0.25 | No | | ns | | | | 0,3616 | |
| 0 vs. 0.5 | No | | ns | | | | 0,5047 | |
| 0 vs. 1 | Yes | | ** | | | | 0,0016 | |
| 0.25 vs. 0.5 | No | | ns | | | | 0,9964 | |
| 0.25 vs. 1 | Yes | | *** | | | | 0,0001 | |
| 0.5 vs. 1 | Yes | | *** | | | | 0,0002 | |
|  |  | |  | | | |  | |
| ***zntA::kan zitB::cam*** | | | | | | |  | |
| 0 vs. 0.25 | Yes | | | **** | | | | <0,0001 |
| 0 vs. 0.5 | Yes | | | **** | | | | <0,0001 |
| 0 vs. 1 | Yes | | | **** | | | | <0,0001 |
| 0.25 vs. 0.5 | No | | | ns | | | | 0,1429 |
| 0.25 vs. 1 | Yes | | | * | | | | 0,0433 |
| 0.5 vs. 1 | No | | | ns | | | | 0,9938 |
| **Within each row, compare columns (simple effects within rows)** | | | | | | | | |
| Tukey's multiple comparisons test | | Significant? | | | Summary | Adjusted P Value | | |
|  | |  | | |  |  | | |
| **-** | |  | | |  |  | | |
| STM vs. *zntA::kan* | | No | | | ns | 0,1121 | | |
| STM vs. *zitB::cam* | | Yes | | | ** | 0,0047 | | |
| STM vs. *zntA::kan zitB::cam* | | No | | | ns | 0,9972 | | |
| *zntA::kan* vs. *zitB::cam* | | Yes | | | **** | <0,0001 | | |
| *zntA::kan* vs. *zntA::kan zitB::cam* | | No | | | ns | 0,1064 | | |
| *zitB::cam* vs. *zntA::kan zitB::cam* | | Yes | | | *** | 0,0008 | | |
|  | |  | | |  |  | | |
| **0.25** | |  | | |  |  | | |
| STM vs. *zntA::kan* | | Yes | | | **** | <0,0001 | | |
| STM vs. *zitB::cam* | | No | | | ns | 0,926 | | |
| STM vs. *zntA::kan zitB::cam* | | Yes | | | **** | <0,0001 | | |
| *zntA::kan* vs. *zitB::cam* | | Yes | | | **** | <0,0001 | | |
| *zntA::kan* vs. *zntA::kan zitB::cam* | | Yes | | | *** | 0,0005 | | |
| *zitB::cam* vs. *zntA::kan zitB::cam* | | Yes | | | **** | <0,0001 | | |
|  | |  | | |  |  | | |
| **0.5** | |  | | |  |  | | |
| STM vs. *zntA::kan* | | Yes | | | **** | <0,0001 | | |
| STM vs. *zitB::cam* | | Yes | | | * | 0,0349 | | |
| STM vs. *zntA::kan zitB::cam* | | Yes | | | **** | <0,0001 | | |
| *zntA::kan* vs. *zitB::cam* | | Yes | | | **** | <0,0001 | | |
| *zntA::kan* vs. *zntA::kan zitB::cam* | | No | | | ns | 0,9969 | | |
| *zitB::cam* vs. *zntA::kan zitB::cam* | | Yes | | | **** | <0,0001 | | |
|  | |  | | |  |  | | |
| **1** | |  | | |  |  | | |
| STM vs. *zntA::kan* | | Yes | | | **** | <0,0001 | | |
| STM vs. *zitB::cam* | | No | | | ns | 0,2974 | | |
| STM vs. *zntA::kan zitB::cam* | | Yes | | | **** | <0,0001 | | |
| *zntA::kan* vs. *zitB::cam* | | Yes | | | **** | <0,0001 | | |
| *zntA::kan* vs. *zntA::kan zitB::cam* | | No | | | ns | 0,998 | | |
| *zitB::cam* vs. *zntA::kan zitB::cam* | | Yes | | | **** | <0,0001 | | |

**S2.B**

| **Within each column, compare rows (simple effects within columns)** | | | |
| --- | --- | --- | --- |
| Tukey's multiple comparisons test | Significant? | Summary | Adjusted P Value |
|  |  |  |  |
| **STM** |  |  |  |
| - vs. 0.25 | No | ns | 0,2835 |
| - vs. 0.5 | No | ns | 0,0737 |
| - vs. 1 | No | ns | 0,8329 |
| 0.25 vs. 0.5 | No | ns | 0,8906 |
| 0.25 vs. 1 | No | ns | 0,7649 |
| 0.5 vs. 1 | No | ns | 0,3461 |
|  |  |  |  |
| ***zntA::kan*** |  |  |  |
| - vs. 0.25 | No | ns | 0,9937 |
| - vs. 0.5 | Yes | **** | <0,0001 |
| - vs. 1 | Yes | **** | <0,0001 |
| 0.25 vs. 0.5 | Yes | **** | <0,0001 |
| 0.25 vs. 1 | Yes | **** | <0,0001 |
| 0.5 vs. 1 | No | ns | 0,9614 |
|  |  |  |  |
| ***zitB::cam*** |  |  |  |
| - vs. 0.25 | No | ns | 0,9505 |
| - vs. 0.5 | No | ns | 0,6103 |
| - vs. 1 | No | ns | 0,6184 |
| 0.25 vs. 0.5 | No | ns | 0,8957 |
| 0.25 vs. 1 | No | ns | 0,9007 |
| 0.5 vs. 1 | No | ns | >0,9999 |
|  |  |  | |
| ***zntA::kan zitB::cam*** | |  | |
| - vs. 0.25 | Yes | **** | <0,0001 |
| - vs. 0.5 | Yes | **** | <0,0001 |
| - vs. 1 | Yes | **** | <0,0001 |
| 0.25 vs. 0.5 | No | ns | 0,9599 |
| 0.25 vs. 1 | No | ns | 0,8184 |
| 0.5 vs. 1 | No | ns | 0,981 |

**Within each row, compare columns (simple effects within rows)**

| Tukey's multiple comparisons test | Significant? | Summary | Adjusted P Value |
| --- | --- | --- | --- |
|  |  |  |  |
| **-** |  |  |  |
| STM vs. *zntA::kan* | Yes | ** | 0,0084 |
| STM vs. *zitB::cam* | No | ns | 0,5146 |
| STM vs. *zntA::kan zitB::cam* | No | ns | 0,0846 |
| *zntA::kan* vs. *zitB::cam* | No | ns | 0,1906 |
| *zntA::kan* vs. *zntA::kan zitB::cam* | No | ns | 0,7649 |
| *zitB::cam* vs. *zntA::kan zitB::cam* | No | ns | 0,7051 |
|  |  |  |  |
| **0.25** |  |  |  |
| STM vs. *zntA::kan* | No | ns | 0,5304 |
| STM vs. *zitB::cam* | No | ns | 0,9996 |
| STM vs. *zntA::kan zitB::cam* | Yes | **** | <0,0001 |
| *zntA::kan* vs. *zitB::cam* | No | ns | 0,5943 |
| *zntA::kan* vs. *zntA::kan zitB::cam* | Yes | **** | <0,0001 |
| *zitB::cam* vs. *zntA::kan zitB::cam* | Yes | **** | <0,0001 |
|  |  |  |  |
| **0.5** |  |  |  |
| STM vs. *zntA::kan* | Yes | **** | <0,0001 |
| STM vs. *zitB::cam* | No | ns | 0,9997 |
| STM vs. *zntA::kan zitB::cam* | Yes | **** | <0,0001 |
| *zntA::kan* vs. *zitB::cam* | Yes | **** | <0,0001 |
| *zntA::kan* vs. *zntA::kan zitB::cam* | No | ns | >0,9999 |
| *zitB::cam* vs. *zntA::kan zitB::cam* | Yes | **** | <0,0001 |
|  |  |  |  |
| **1** |  |  |  |
| STM vs. *zntA::kan* | Yes | **** | <0,0001 |
| STM vs. *zitB::cam* | No | ns | 0,3076 |
| STM vs. *zntA::kan zitB::cam* | Yes | **** | <0,0001 |
| *zntA::kan* vs. *zitB::cam* | Yes | **** | <0,0001 |
| *zntA::kan* vs. *zntA::kan zitB::cam* | No | ns | 0,9999 |
| *zitB::cam* vs. *zntA::kan zitB::cam* | Yes | **** | <0,0001 |

# Supplementary Figure S1

Growth curves of STM, *zntA::kan*, *zitB::cam* and *zntA::kan zitB::cam* strains in VBMM with increasing concentration of ZnSO_4_, as indicated in the legend.


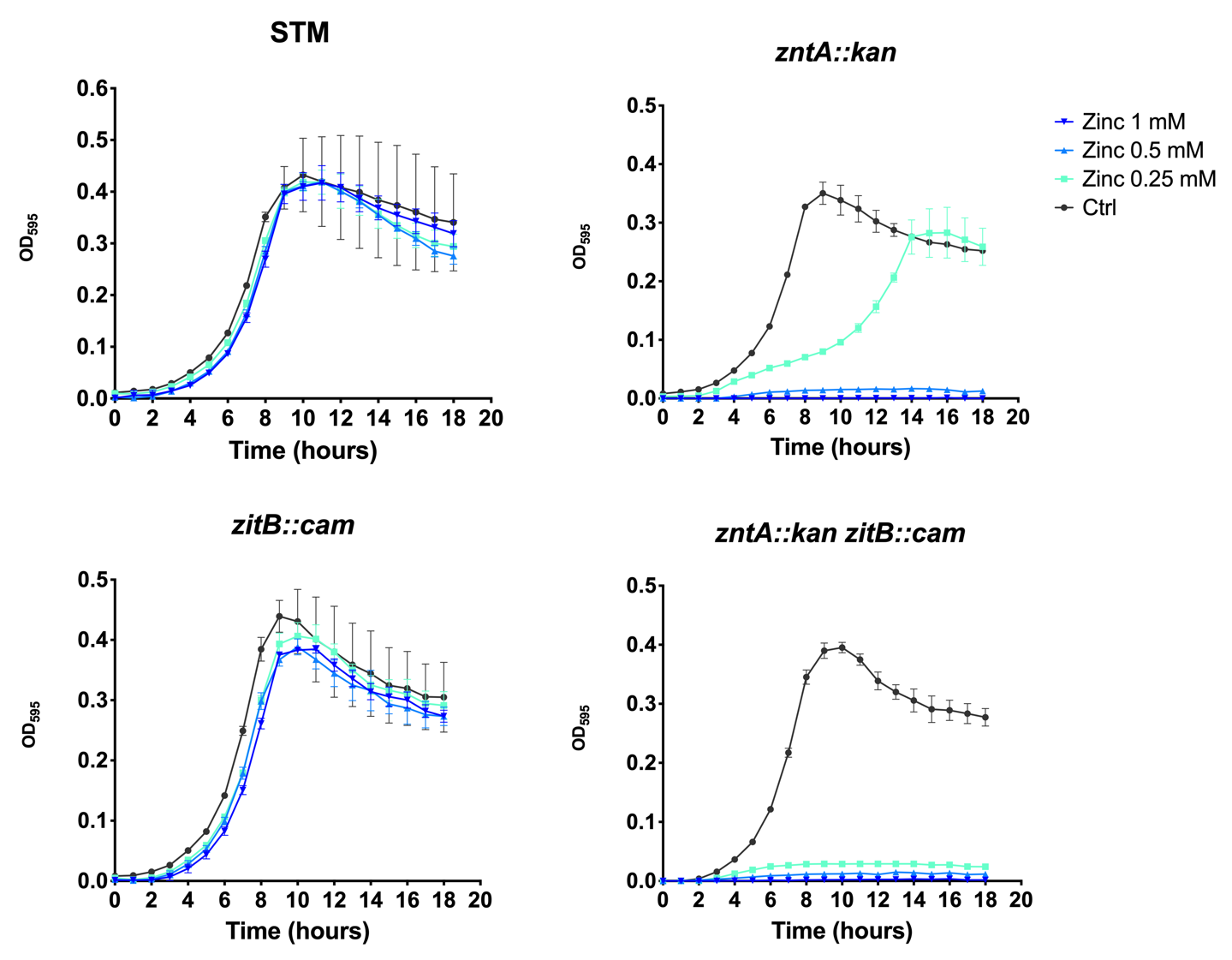

Supplement: Supplementary file 1 [file Data_Sheet_1.docx]
